# Factors associated with medical device-related pressure injury in an intensive care unit in the Amazon region during the COVID-19 pandemic: retrospective cohort

**DOI:** 10.3389/fmed.2025.1605584

**Published:** 2025-07-23

**Authors:** Vitória Alice Alencar Sousa, Daniele Nunes da Silva Ferreira, Giovana Vitória Guimarães Mendonça, Rebecca Lobato Marinho, Natasha Cristina Oliveira Andrade, Tamires de Nazaré Soares, Marcos Jessé Abrahão Silva, Diana da Costa Lobato, Suziane do Socorro dos Santos, Yan Corrêa Rodrigues, Daniele Melo Sardinha, Luana Nepomuceno Gondim Costa Lima

**Affiliations:** ^1^Secretaria Municipal de Saúde (SESAU), Benevides, Brazil; ^2^Programa de Pós-Graduação em Biologia Parasitária na Amazônia, Universidade do Estado do Pará and Instituto Evandro Chagas (PPGBPA/UEPA/IEC), Belém, Brazil; ^3^Seção de Bacteriologia e Micologia, Instituto Evandro Chagas (SABMI/IEC), Ananindeua, Brazil; ^4^Programa de Pós-Graduação em Epidemiologia e Vigilância em Saúde, Instituto Evandro Chagas (PPGEVS/IEC), Ananindeua, Brazil

**Keywords:** epidemiology, pressure ulcer medical, medical devices, adult intensive care units, associated factor

## Abstract

Medical device–related pressure injuries (MDRPIs) pose a serious public health challenge, particularly in intensive care settings during the COVID-19 pandemic. This retrospective cohort study aimed to characterize the profile and identify risk and protective factors for MDRPIs among adult ICU patients in a metropolitan region of the Amazon between January 2021 and December 2022. We reviewed 603 medical records—31 patients (5.1%) developed MDRPIs and 572 did not—and applied chi-square tests, normality assessments, Mann–Whitney U-tests, binary logistic regression, and Kaplan–Meier survival analysis. Ethical approval was obtained from the Fundação Hospital de Clínicas Gaspar Viana Ethics Committee (approval no. 5,991,542). Independent risk factors for MDRPIs included chronic obstructive pulmonary disease (OR 19.33; 95% CI 2.92–127.73; *p* = 0.002), orotracheal tube use (OR 19.00; p = 0.002), nasal catheter use (OR 3.33; 95% CI 1.32–8.40; *p* = 0.011), and longer hospital stay (OR 1.09 per day; 95% CI 1.05–1.12; *p* < 0.001). Protective factors were systemic arterial hypertension (OR 0.22; 95% CI 0.08–0.58; *p* = 0.009), higher Braden scale scores (OR 0.22 per point; 95% CI 0.08–0.58; *p* = 0.002), and invasive arterial blood pressure monitoring (OR 0.14; 95% CI 0.03–0.79; *p* = 0.025). Survival analysis demonstrated that patients with MDRPIs had significantly longer hospital stays and higher mortality rates (Breslow *p* = 0.007; log-rank *p* = 0.041; Tarone–Ware *p* = 0.011). This first study of MDRPIs in the Amazon region highlights key modifiable factors and underscores the need for enhanced nursing protocols and working conditions to prevent device-related pressure injuries in critical care. These findings can guide continuing education initiatives and policy development in critical care nursing.

## 1 Introduction

Medical device-related pressure injuries (MDRPIs) constitute a significant public health concern (1). The etiology of these injuries is multifactorial, involving damage to integumentary tissues caused by prolonged pressure. The design, configuration, and positioning of medical devices used for therapeutic purposes significantly influence the development of these injuries (2–4). Unfortunately, MDRPIs have become increasingly common in hospital settings, particularly among vulnerable patients admitted to Intensive Care Units (ICUs) (5). MDRPIs have a dual impact: they cause physical and psychological distress for patients and impose substantial costs on healthcare institutions and patients’ families. They have been associated with increased morbidity and mortality rates and are considered indicators of suboptimal healthcare quality (6).

The COVID-19 pandemic has exacerbated the incidence of MDRPIs due to increased ICU admissions and weakened healthcare services (7). Consequently, there has been a heightened use of medical devices in critical care, further contributing to MDRPI prevalence (2).

Existing literature highlights that ICU patients are especially susceptible to MDRPIs due to risk factors such as prolonged hospital stays, frequent use of medical devices, and hemodynamic instability related to medication use (8, 9). Additionally, MDRPIs can result from healthcare professionals prioritizing the treatment of acute illnesses or other clinical conditions over comprehensive skin care (8, 10). Notably, incorrect device use, inadequate positioning or securing of equipment, improper selection of medical devices, insufficient skin assessment, and device malfunction can significantly increase the risk of MDRPIs (11).

In Brazil, the National Health Surveillance Agency (ANVISA) maintains the Health Surveillance Notification system (NOTIVISA), enabling the reporting of adverse events, including MDRPIs, and technical complaints in hospitalized patients. Data collected via NOTIVISA support the implementation of measures aimed at health protection and promotion (12). However, surveillance efficacy is compromised due to incomplete reporting forms, resulting in underreporting and a lack of accurate data.

Currently, no studies have been conducted in the Brazilian Amazon region to investigate the profile and associated factors of ICU patients affected by MDRPIs. Given their clinical characteristics, these patients are particularly vulnerable to injuries from medical devices.

This study aims to identify the patient profile and associated factors related to medical device-induced pressure injuries in an adult ICU within a metropolitan area of the Amazon during the COVID-19 pandemic.

## 2 Materials and methods

### 2.1 Type of study and ethical aspects

This study is a retrospective longitudinal cohort analysis based on data from physical records, documents, and free-text notes obtained from a reference hospital in the region. Data collection occurred over 3 months, from April to June 2023. The sample included medical records of patients admitted to the adult ICU at the reference hospital who utilized medical devices during their hospitalization.

The study adhered to the guidelines of the Strengthening the Reporting of Observational Studies in Epidemiology (STROBE) statement (13).

Ethical protocols were strictly followed to ensure the confidentiality of the collected data and protect participants from potential harm. Ethical approval was granted by the Research Ethics Committee of Fundação Hospital de Clínicas Gaspar Viana (approval number: 5,991,542), in compliance with Resolution 196/96 of the Brazilian National Health Council.

### 2.2 Selection of participants

**Figure 1:**
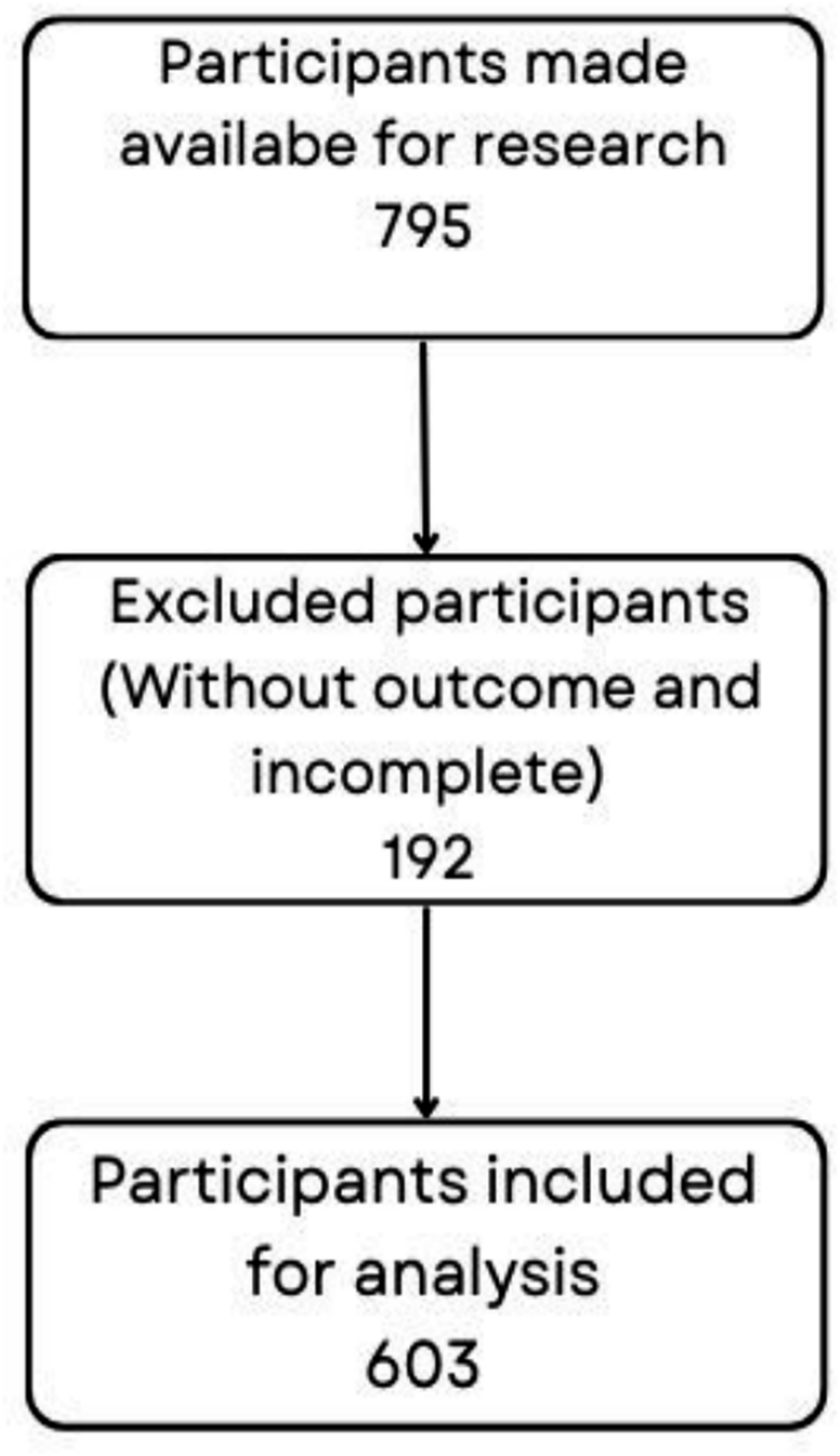
Participant selection flowchart.

The study included adult patients admitted to ICUs I and II who utilized medical devices during their hospitalization. Inclusion criteria comprised complete, legible, and finalized medical records of patients aged 18 years or older, of both sexes, admitted between 1 January 2021 and 31 December 2022. Medical records that were incomplete, illegible, or lacked conclusions were excluded. The patient selection process leading to the final study population is depicted in the flowchart below (Figure 1).

### 2.3 MDRPI case definition

In 2016, the National Pressure Ulcer Advisory Panel updated its definition of pressure injury (PI), describing it as localized damage to the skin and/or underlying soft tissue, typically over a bony prominence or related to the use of a medical device or another object. Medical device-related pressure injuries (MDRPIs) usually adopt the shape of the device involved and should be classified according to the existing PI classification system (3).

### 2.4 Data collection

The data collection process involved transcribing information from the medical record into an assessment form with pre-formulated questions. This form served as a guide for the research.

The eligibility criteria were based on the selection of cases in adult ICUs I and II. The cohort’s time zero was defined by hospitalization data, and the delta-time (∆T) corresponded to the period from hospitalization data to the stage of cure or death for those hospitalized. The time sequence continued until the end.

Subsequently, the data were organized in Excel 2019 format with 60 variables added to the form, referring to sociodemographic and clinical-epidemiological data. The variables extracted from the form were: alphanumeric medical record number (item 1), age (item 2), sex (item 3), length of stay (item 4), pulmonary arterial hypertension (item 5), systemic arterial hypertension (SAH) (item 6), diabetes mellitus (DM) (item 7), obesity (item 8), heart disease (item 9), congestive heart failure (CHF) (item 10), acute myocardial infarction (AMI) (item 11), and arrhythmia (item 12). The following medical conditions are listed: acute renal failure (ARF) (item 13), chronic kidney disease (CKD) (item 14), autoimmune disease (item 15), stroke (item 16), cancer (CA) (item 17), multimorbidities (item 18), infectious disease (item 19), multi-diseases (item 20), alcoholism (item 21), former alcoholic (item 22), smoker (item 23), former smoker (item 24), and allergy to medication (item 25). The patient has reported allergy to product (item 26), pulmonary arterial obstructive disease (PAOD) (item 27), osteoporosis (item 28), pneumopathy (item 29), hypothyroidism (item 30), hyperthyroidism (item 31), benign prostatic hyperplasia (item 32), Down syndrome (item 33), chronic obstructive pulmonary disease (COPD) (item 34), and asthma (item 35), as well as anxiety (item 36). The following medical conditions are mentioned in the text: schizophrenia (item 37), gastritis (item 38), drug use (item 39), endocrinopathy (item 40), cirrhosis (item 41), rheumatic fever (item 42), Chagas disease (item 43), hepatitis B (item 44), hepatitis C (item 45), Human Immunodeficiency Virus (HIV) (item 46), Herpes (item 47), Syphilis (item 48), tuberculosis (TB) (item 49), and *Klebsiella pneumoniae* carbapenemase (KPC) (item 50), The following items were mentioned in the text: streptococcus maltophilia (item 51), invasive ventilation (item 52), monitoring of pressure sources and skin friction (item 53), Braden scale (item 54), peripheral venous access (PVA) (item 55), Nasogastric Tube (NGT) (item 56), Central Venous Catheter (CVC) (item 57), Orotracheal Tube (OTT) (item 58), Bladder Indwelling Probe (BIP) (item 59), and Bladder Relief Probe (BRP) (item 60). The following medical items were used: Nasal Catheter (NC) (item 61), Nasoenteral Tube (ENS) (item 62), Double-Lumen Catheter (DLM) (item 63), drain (item 64), macro nebulization (item 65), Tracheostomy (TQT) (item 66), Pulse Oximeter (PO) (item 67), Invasive Blood Pressure (IBP) (item 68), Non-Rebreathing Mask (NRM) (item 69), pacemaker (item 70), and Ventricular Arterial Shunt (VAS) (item 71). The patient’s condition was assessed based on several factors including peripheral perfusion (items 72 and 73), urinary stream (item 74), anuria (item 75), oliguria (item 76), complications during hospitalization (item 77), type of complication (item 78), urinary elimination (items 79–81), hematuria (item 82), lumps (item 83), and urinary urgency (item 84). The following items are included in this list: ureteroileostomy (item 85), absent intestinal elimination (item 86), normal intestinal elimination (item 87), elimination of intestinal diarrhea (item 88), hematochezia (item 89), melena (item 90), and constipation (item 91). The list aims to provide a comprehensive overview of mobility levels: room air (item 92), oxygen therapy (item 93), intubated (item 94), tracheostomized (item 95), conscious (item 96), oriented (item 97), disoriented (item 98), torpid (item 99), sedated (item 100), and agitated (item 101). The patient’s condition is described as follows: item 102 indicates that they are bedridden, item 103 indicates that they walk with assistance, item 104 indicates that they are restricted to bed, and item 105 indicates that they are able to walk. Additional information includes the patient’s oximetry value (item 114), Braden scale score (item 115), and number of medications taken during hospitalization (item 116).

The Braden Scale is a tool used in intensive care units to assess the risk of pressure injuries in critical patients. Nurses use this tool to record the patient’s risk level and take preventative measures to promote effective treatment and greater patient comfort. The scale consists of six subscales for assessing six variables: Sensory Perception, Moisture, Activity, Mobility, Nutrition, and Friction and Shear. The subscales are scored on a scale of 1 to 4, with the exception of the Friction and Shear variable, which is scored on a scale of 1 to 3.

### 2.5 Statistical procedures

This study compared patients with MDRPI (exposed group) and without MDRPI (unexposed group). The comparative analyses included patient demographics, presence of comorbidities, hospitalization characteristics, types of medical devices, and lesion stages in both groups.

Data were organized in Microsoft Excel (version 2019) and analyzed using Jamovi software (version 2.3.28). Chi-square and Fisher’s exact tests (L × C contingency table) were performed to evaluate the independence between categorical variables. Odds ratios (ORs) and their corresponding confidence intervals were calculated for variables with statistical significance (*p* ≤ 0.05), comparing the MDRPI and non-MDRPI groups. Results were summarized and presented in tables.

Numerical variables underwent the Kolmogorov–Smirnov test to evaluate normality and were subsequently analyzed using the Mann–Whitney *U*-test due to their non-parametric distribution.

Binary logistic regression analysis was conducted to determine the relationship between MDRPI (dependent variable) and various independent variables. Both univariate and multivariate regression models were employed to identify factors significantly associated with MDRPI development.

Survival analysis was performed using the Kaplan–Meier method, considering the time from the initiation of hospitalization to either discharge or death, and accounting for variations related to device type, hospitalization reason, comorbidities, and treatment type. These outcomes were further analyzed with the Statistical Package for the Social Sciences (SPSS). The statistical significance level adopted for all analyses was set at 0.05.

## 3 Results

This study analyzed 603 medical records to identify the profile and factors associated with Medical Device-Related Pressure Injuries (MDRPI) among patients admitted to the Intensive Care Unit (ICU) of a metropolitan hospital during 2021 and 2022. Out of these records, 31 patients (5.14%) developed MDRPI, while 572 patients (94.86%) did not.

Initially, the epidemiological profile of the sample was characterized through bivariate analysis of 109 categorical variables. Of the total patients, 62.85% (379) were female, and 37.15% (224) were male. Among the sample, 15.26% (92) had medication allergies, and 0.50% (3) had product allergies. Regarding substance use, 14.59% (88) were alcoholics, and 23.71% (143) were former alcoholics. Additionally, 13.60% (82) were smokers, and 34.83% (210) were former smokers.

The epidemiological characteristics of the 603-patient cohort revealed that 219 individuals (36.32%) had diabetes mellitus and 449 (74.46%) had systemic arterial hypertension. Acute myocardial infarction was present in 12 patients (1.99%), heart disease in 89 (14.76%), pulmonary obstructive arterial disease in 1 (0.17%), osteoporosis in 5 (0.83%), hypo- or hyperthyroidism in 6 (1.00%), pulmonary arterial hypertension in 1 (0.17%), benign prostatic hyperplasia in 1 (0.17%), and Down syndrome in 1 (0.17%). Other comorbidities included chronic obstructive pulmonary disease (COPD; 17.00%), acute renal failure (1.49%), chronic heart failure (5.64%), asthma (1.00%), chronic kidney disease (7.30%), stroke (0.33%), arrhythmia (1.82%), anxiety (0.50%), schizophrenia (0.17%), obesity (1.82%), gastritis (0.17%), cancer (1.49%), autoimmune disease (0.66%), endocrinopathy (0.17%), cirrhosis (0.33%), rheumatic fever (0.17%), and multimorbidity (65.67%).

Table 1 shows the incidence of infectious conditions: other infectious diseases in 26 patients (4.31%), Chagas disease in 7 (1.16%), hepatitis B in 4 (0.66%), hepatitis C in 1 (0.17%), HIV in 9 (1.49%), herpes in 4 (0.66%), syphilis in 2 (0.33%), tuberculosis in 1 (0.17%), carbapenem-resistant *Klebsiella pneumoniae* (KPC) in 1 (0.17%), *Stenotrophomonas maltophilia* in 1 (0.17%), and multiple concurrent infections in 3 (0.50%). In the bivariate comparison between MDRPI carriers and non-carriers, systemic arterial hypertension (*p* < 0.01; OR 0.298; 95% CI 0.144–0.619), COPD (*p* = 0.019; OR 5.57; 95% CI 1.11–28.0), and multimorbidity (*p* = 0.003; OR 5.17; 95% CI 1.55–17.2) were significantly associated with the occurrence of MDRPI.

**Table 1:** Bivariate model for epidemiological factors associated with MDRPI in patients hospitalized in an intensive care unit of a hospital in the Brazilian Amazon in 2021 and 2022.

Epidemiological variables	Without MDRPI 572	%	With MDRPI 31	%	Total 603	%	*p*-value	OR	CI 95%	
Masculine	212	37.06	12	38.71	224	37.15	*0.813			
Drug allergy	86	15.03	6	19.35	92	15.26	*0.515			
Product allergy	3	0.52	0	0.00	3	0.50	¨0.686			
Alcoholism	85	14.86	3	9.68	88	14.59	¨0.426			
Former alcoholic	137	23.95	6	19.35	143	23.71	*0.558			
Smoker	80	13.99	2	6.45	82	13.60	¨0.233			
Former smoker	197	34.44	13	41.94	210	34.83	*0.394			
Diabetes mellitus	209	36.54	10	32.26	219	36.32	*0.629			
Hypertension	434	75.87	15	48.39	449	74.46	* < 0.001	0.298	0.144	0.619
AMI	12	2.10	0	0.00	12	1.99	¨0.415			
Cardiopathy	86	15.03	3	9.68	89	14.76	¨0.413			
PAOD	1	0.17	0	0.00	1	0.17	¨0.816			
Osteoporosis	5	0.87	0	0.00	5	0.83	¨0.801			
Pneumopathy	1	0.17	0	0.00	1	0.17	¨0.816			
Hypothyroidism	6	1.05	0	0.00	6	1.00	¨0.567			
Hyperthyroidism	1	0.17	0	0.00	1	0.17	¨0.816			
Pulmonary arterial hypertension	1	0.17	0	0.00	1	0.17	¨0.816			
Benign prostatic hyperplasia	1	0.17	0	0.00	1	0.17	¨0.816			
Down’s syndrome	1	0.17	0	0.00	1	0.17	¨0.816			
COPD	7	1.22	2	6.45	9	1.49	¨0.019	5.57	1.11	28
ARF	1	0.17	0	0.00	1	0.17	¨0.816			
CHF	31	5.42	3	9.68	34	5.64	¨0.317			
Asthma	6	1.05	0	0.00	6	1.00	¨0.567			
CKD	40	6.99	4	12.90	44	7.30	¨0.218			
Stroke	2	0.35	0	0.00	2	0.33	¨0.742			
Arrhythmia	11	1.92	0	0.00	11	1.82	¨0.436			
Anxiety	3	0.52	0	0.00	3	0.50	¨0.686			
Schizophrenia	1	0.17	0	0.00	1	0.17	¨0.816			
Obesity	10	1.75	1	3.23	11	1.82	¨0.549			
Gastritis	1	0.17	0	0.00	1	0.17	¨0.816			
Here	9	1.57	0	0.00	9	1.49	¨0.482			
Autoimmune disease	4	0.70	0	0.00	4	0.66	¨0.64			
Drug user	6	1.05	0	0.00	6	1.00	¨0.567			
Endocrinopathy	1	0.17	0	0.00	1	0.17	¨0.816			
Cirrhosis	2	0.35	0	0.00	2	0.33	¨0.742			
Rheumatic fever	1	0.17	0	0.00	1	0.17	¨0.816			
Multimorbidities	368	64.34	28	90.32	396	65.67	*0.003	5.17	1.55	17.2
Infectious disease	25	4.37	1	3.23	26	4.31	¨0.76			
Chagas	7	1.22	0	0.00	7	1.16	¨0.536			
Hepatitis B	4	0.70	0	0.00	4	0.66	¨0.64			
Hepatitis C	1	0.17	0	0.00	1	0.17	¨0.816			
HIV	9	1.57	0	0.00	9	1.49	¨0.482			
Herpes	4	0.70	0	0.00	4	0.66	¨0.64			
Syphilis	1	0.17	1	3.23	2	0.33	¨0.004	19.0	1.16	312
Also	1	0.17	0	0.00	1	0.17	¨0.816			
KPC	1	0.17	0	0.00	1	0.17	¨0.816			
Maltophilia streptococcus	1	0.17	0	0.00	1	0.17	¨0.816			
Multidiseases	3	0.52	0	0.00	3	0.50	¨0.686			

Source: Authors’ research. The chi-square test, for values less than 5, or Fisher’s exact test was used. *The chi-square. ¨ Fisher’s.

Table 2 summarizes hospitalization-related variables. Invasive mechanical ventilation was required by 49.42% of patients, while pressure-and-friction monitoring and Braden scale assessments were each performed in 59.54% of cases. The most frequently used medical devices were indwelling bladder catheters (*n* = 378; 62.69%), peripheral venous access (*n* = 347; 57.55%), orotracheal tubes (*n* = 301; 49.92%), nasoenteral tubes (*n* = 285; 47.26%), and central venous catheters (*n* = 257; 42.62%). Additional devices included double-lumen catheters (26%), surgical drains (*n* = 187; 31.01%), macronebulization (*n* = 51; 8.46%), tracheostomies (*n* = 41; 6.80%), pulse oximeters (*n* = 72; 11.94%), invasive arterial pressure monitors (*n* = 76; 12.60%), non-rebreathing masks (*n* = 5; 0.83%), pacemakers (*n* = 4; 0.66%), and vasoactive drug infusions (*n* = 7; 1.16%).

**Table 2:** Bivariate model for hospitalization factors associated with MDRPI in patients hospitalized in an intensive care unit of a hospital in the Brazilian Amazon in 2021 and 2022.

International variables	Without MDRPI 572	%	With MDRPI 31	%	Total 603	%	*p*-value	OR	CI 95%	
Invasive ventilation	278	48.60	20	64.52	298	49.42	*0.084			
Monitoring sources of skin pressure and friction	348	60.84	11	35.48	359	59.54	*0.005	0.354	0.166	0.753
Performed Braden scale	348	60.84	11	35.48	359	59.54	*0.005	0.354	0.166	0.753
Peripheral venous access	332	58.04	15	48.39	347	57.55	¨0.289			
Nasogastric tube	34	5.94	0	0.00	34	5.64	¨0.162			
Central venous catheter	239	41.78	18	58.06	257	42.62	*0.074			
Orotracheal tube	274	47.90	27	87.10	301	49.92	* < 0.001	7.34	2.54	21.2
Indwelling bladder catheter	353	61.71	25	80.65	378	62.69	*0.034	2.58	1.04	6.4
Bladder relief probe	6	1.05	0	0.00	6	1.00	¨0.567			
Nasal catheter	124	21.68	11	35.48	135	22.39	*0.072			
Nasoenteral tube	259	45.28	26	83.87	285	47.26	* < 0.001	6.28	2.38	16.6
Double lumen catheter	174	30.42	13	41.94	187	31.01	*0.177			
Drain	48	8.39	3	9.68	51	8.46	¨0.802			
Macronebulization	5	0.87	0	0.00	5	0.83	¨0.601			
Tracheostomy	33	5.77	8	25.81	41	6.80	* < 0.001	5.68	2.36	13.7
Pulse oximeter	72	12.59	0	0.00	72	11.94	¨0.035	0.110	0.00663	1.81
Invasive blood pressure	74	12.94	2	6.45	76	12.60	¨0.289			
Non-rebreathing mask	5	0.87	0	0.00	5	0.83	¨0.601			
Pacemaker	4	0.70	0	0.00	4	0.66	¨0.64			
Vasoactive drugs	7	1.22	0	0.00	7	1.16	¨0.536			
Good peripheral perfusion	380	66.43	10	32.26	390	64.68	* < 0.001	0.241	0.111	0.521
Decreased peripheral perfusion	158	27.62	15	48.39	173	28.69	*0.013	2.46	1.19	5.09
Decreased urinary stream	40	6.99	2	6.45	42	6.97	¨0.908			
Anuria	130	22.73	4	12.90	134	22.22	¨0.2			
Oliguria	2	0.35	0	0.00	2	0.33	¨0.742			
Normal urinary elimination	347	60.66	19	61.29	366	60.70	*0.945			
Choluric urinary elimination	5	0.87	0	0.00	5	0.83	¨0.601			
Elimination of urinary cystostomy	2	0.35	3	9.68	5	0.83	¨ < 0.001	30.5	4.90	190
Hematuria	17	2.97	1	3.23	18	2.99	¨0.936			
Groats	1	0.17	0	0.00	1	0.17	¨0.816			
Urinary urgency	1	0.17	0	0.00	1	0.17	¨0.816			
Cutaneous ureteroileostomy	1	0.17	0	0.00	1	0.17	¨0.816			
Intestinal absent elimination	445	77.80	17	54.84	462	76.62	*0.003	0.347	0.166	
Normal intestinal elimination	64	11.19	7	22.58	71	11.77	*0.055	2.32	0.959	5.59
Elimination of intestinal diarrhea	16	2.80	3	9.68	19	3.15	¨0.033	3.72	1.02	13.5
Hematochezia	1	0.17	0	0.00	1	0.17	¨0.816			
Melena	1	0.17	0	0.00	1	0.17	¨0.816			
Constipation	13	2.27	1	3.23	14	2.32	¨0.713			
Ambient air	206	36.01	4	12.90	210	34.83	¨0.009	0.263	0.908	0.763
Oxygen therapy	135	23:60	6	19.35	141	23.38	*0.586			
Intubated	215	37.59	21	67.74	236	39.14	* < 0.001	3.49	1.61	7.54
Tracheostomized	16	2.80	0	0.00	16	2.65	¨0.345			
Conscious	36	6.29	10	32.26	46	7.63	*0.012	0.386	0.178	0.834
Oriented	195	34.09	4	12.90	199	33.00	¨0.015	0.286	0.0988	0.83
Disoriented	11	1.92	0	0.00	11	1.82	¨0.436	0.775	0.0446	13.5
Torporous	49	8.57	1	3.23	50	8.29	¨0.294			
Sedated	216	37.76	19	61.29	235	38.97	*0.009	2.61	1.24	5.48
Hectic	3	0.52	1	3.23	4	0.66	¨0.071	6.32	0.638	62.6
Mobility level: bedridden	145	25:35	13	41.94	158	26:20	*0.048	0.316	0.0945	1.05
Mobility level: Walks with assistance	2	0.35	0	0.00	2	0.33	¨0.742			
Mobility level: restricted to bed	413	72.20	28	90.32	441	73.13	*0.028	3.57	1.07	11.9
Mobility level: walks	3	0.52	0	0.00	3	0.50	¨0.686			
Overweight	38	6.64	4	12.90	42	6.97	¨0.182			
Obesity	55	9.62	7	22.58	62	10.28	*0.021	2.74	1.13	6.65
Weight loss report	35	6.12	3	9.68	38	6.30	¨0.427			
Low weight	5	0.87	0	0.00	5	0.83	¨0.601			
Malnutrition	79	13.81	6	19.35	85	14.10	*0.388			
Normoweight	168	29:37	6	19.35	174	28.86	*0.231			
Hypocaloric	364	63.64	21	67.74	385	63.85	*0.643			
Normocolored	186	32.52	6	19.35	192	31.84	*0.125			
Jaundice	11	1.92	1	3.23	12	1.99	¨0.613			

Source: Authors’ research. The chi-square test, for values less than 5, or Fisher’s exact test was used. *The chi-square. ¨ Fisher’s.

Physiological assessments showed that 390 patients (64.68%) had adequate peripheral perfusion, whereas 173 (28.69%) had decreased perfusion. Urinary output patterns were normal in 366 (60.70%), anuric in 134 (22.22%), decreased stream in 42 (6.97%), oliguria in 2 (0.33%), choluric output in 5 (0.83%), and via cystostomy in 5 (0.83%). Hematuria occurred in 83 patients (13.76%), urinary urgency in 1 (0.17%), and ureteroileostomy in 1 (0.17%). Intestinal elimination was absent in 462 (76.62%), normal in 71 (11.77%), diarrheal in 19 (3.15%), hematochezia in 1 (0.17%), melena in 1 (0.17%), and constipation in 14 (2.32%).

At assessment, 210 patients (34.83%) breathed room air, 141 (23.38%) received supplemental oxygen, 236 (39.14%) were intubated, and 16 (2.65%) were tracheostomized. Neurologically, 46 (7.63%) were fully conscious, 199 (33.00%) oriented, 11 (1.82%) disoriented, 50 (8.29%) drowsy, 235 (38.97%) sedated, and 4 (0.66%) agitated. Mobility status showed 158 patients (26.20%) bedridden, 2 (0.33%) ambulating with assistance, 441 (73.13%) restricted to bed, and 3 (0.50%) walking independently. Nutritional status was normal in 28.86%, obese in 10.28%, overweight in 6.97%, experiencing weight loss in 6.30%, underweight in 0.83%, and malnourished in 14.10% of patients. Skin assessment revealed normal coloration in 31.84%, jaundice in 1.99, and 63.85% were classified as hypocaloric.

When comparing MDRPI carriers and non-carriers, bivariate analysis identified several significant associations. Monitoring of pressure and friction sources (*p* = 0.005; OR = 0.354; 95% CI, 0.166–0.753) and Braden scale assessments (*p* = 0.005; OR = 0.354; 95% CI, 0.166–0.753) were both protective. In contrast, use of an orotracheal tube (*p* < 0.001; OR = 7.34; 95% CI, 2.54–21.20), an indwelling bladder catheter (*p* < 0.001), a nasoenteral tube (*p* < 0.001; OR = 6.28; 95% CI, 2.38–16.60), and tracheostomy (*p* < 0.001; OR = 5.68; 95% CI, 2.36–13.70) were all associated with increased MDRPI risk. Pulse oximetry monitoring also appeared protective (*p* = 0.035; OR = 0.110; 95% CI, 0.00663–1.81).

Among physiological parameters, good peripheral perfusion reduced MDRPI risk (*p* < 0.001; OR = 0.241; 95% CI, 0.111–0.521), whereas decreased perfusion did not reach statistical significance (*p* = 0.13; OR = 2.46; 95% CI, 1.19–5.09). Urinary elimination via cystostomy was significantly linked to MDRPI (*p* < 0.001), and diarrheal elimination increased risk (*p* = 0.033; OR = 3.72; 95% CI, 1.02–13.5), while normal intestinal elimination showed only a marginal association (*p* = 0.055; OR = 2.32; 95% CI, 0.959–5.59). Breathing room air was protective (*p* = 0.009; OR = 0.263; 95% CI, 0.908–0.763), whereas intubation increased risk (*p* < 0.001; OR = 3.49; 95% CI, 1.61–7.54). Finally, patient consciousness and mobility influenced MDRPI risk: oriented patients had lower risk (*p* = 0.015; OR = 0.286; 95% CI, 0.0988–0.830), sedated patients had higher risk (p = 0.009; OR = 2.61; 95% CI, 1.24–5.48), bed mobility was protective (*p* = 0.048; OR = 0.316; 95% CI, 0.0945–1.05), restriction to bed increased risk (*p* = 0.028; OR = 3.57; 95% CI, 1.07–11.9), and obesity was also a risk factor (*p* = 0.021; OR = 2.74; 95% CI, 1.74–6.65).

Mann–Whitney U-tests were applied to all continuous variables. Age did not differ significantly between MDRPI carriers and non-carriers (*p* = 0.131). The overall mean age was 63 ± 15.6 years (median 66; range 18–105). Among the 31 patients with MDRPI, mean age was 67 ± 15.3 years (median 70), and among those without MDRPI, it was 67 ± 15.3 years (median 70; range 28–93).

Length of stay ranged from 1 to 81 days (mean ± SD: 8 ± 9 days; median 5) and was significantly longer in patients who developed MDRPI (mean ± SD: 21 ± 18 days; median 20; range 5–81) than in those who did not (mean ± SD: 7 ± 6 days; median 5; range 1–62; *p* < 0.001), indicating that prolonged hospitalization increases MDRPI risk.

Oxygen saturation averaged 95% ± 3.9 (median 96%; range 55–100%) across all patients and did not differ significantly between groups (*p* = 0.515). In the MDRPI group, saturation values ranged from 74 to 100% (mean 82%; median 95%), whereas in the non-MDRPI group, they ranged from 55 to 100% (mean 92%; median 96%).

Braden scale scores ranged from 0 to 26 points (mean ± SD: 8 ± 7; median: 9). Among patients who developed MDRPI, scores ranged from 0 to 15 (mean ± SD: 3 ± 5; median: 0), compared with a range of 0 to 26 (mean ± SD: 8 ± 7; median: 9) in those without MDRPI (*p* < 0.001), confirming the Braden scale’s predictive value in skin assessment and injury prevention.

The number of medications administered during hospitalization (Table 3) varied from 0 to 50. Patients who developed MDRPI received significantly more medications (range: 7–50; mean ± SD: 21 ± 11; median: 18) than those without MDRPI (range: 0–50; mean ± SD: 13 ± 8; median: 12; *p* < 0.001), indicating an association between polypharmacy and MDRPI risk.

**Table 3:** Bivariate model for numerical variables associated with MDRPI in patients hospitalized in an intensive care unit of a hospital in the Brazilian Amazon in 2021 and 2022.

Numerical factors	Total (603)	MDRPI (31)	Without MDRPI (572)	*p*-value
Age				
Minimum Maximum	18–105	28–93	18–105	
Media	63.71	67.12	63.67	
Median	66	70	66	0.131
Standard deviation	15,634	15.34	15.61	
Length of stay				
Minimum Maximum	55–100	74–100	55–100	
Media	8.78	21.06	7.93	
Median	5.00	20	5	<0.001
Standard deviation	9,678	16.27	8.52	
Oximetry value				
Minimum Maximum	55–100	74–100	55–100	
Media	95.51	82.51	92.37	
Median	96	95	96	0.515
Standard deviation	3.99	32.63	17.56	
Braden scale score				
Minimum Maximum	0–26	0–15	0–26	
Media	8,538	3,677	8,530	
Median	9.00	0	9	<0.001
Standard deviation	75,628	5,224	7,607	
Number of medications during hospitalization				
Minimum Maximum	0–50	7–50	0–50	
Media	14.33	21.90	13.94	
Median	12	18	12	<0.001
Standard deviation	8,906	11.61	8,658	

Source: Table created in Excel; Statistical test carried out in the Statistical Package for Social Science for Windows (SPSS).

Table 4 presents the multivariate logistic regression of factors independently associated with MDRPI. Chronic obstructive pulmonary disease emerged as the strongest risk factor (*p* = 0.002; OR 19.33; 95% CI 2.92–127.73), followed by orotracheal tube use (*p* = 0.002; OR 19.00; 95% CI –), nasal catheter use (*p* = 0.011; OR 3.33; 95% CI 1.32–8.40), and longer hospital stay (*p* < 0.001; OR 1.09; 95% CI 1.05–1.12). Protective predictors included systemic arterial hypertension (*p* = 0.009; OR 0.22; 95% CI 0.08–0.58), higher Braden scale scores (*p* = 0.002; OR 0.22; 95% CI 0.08–0.58), and invasive arterial pressure monitoring (*p* = 0.025; OR 0.14; 95% CI 0.27–0.79).

**Table 4:** Final multivariate model for associated factors for MDRPI in patients hospitalized in an intensive care unit of a hospital in the Brazilian Amazon in 2021 and 2022.

Variables	*p*-value	OR	CI 95%	
			Minimum	Maximum
Chronic obstructive pulmonary disease	0.002	19.3256	2.92404	127.7263
Orotracheal tube	0.004	5.4812	1.70836	17.5865
Nasal Catheter	0.011	3.3297	1.31973	8.4008
Length of stay	<0.001	1.0863	1.05026	1.1236
Systemic arterial hypertension	0.009	0.3155	0.13198	0.7543
Braden scale score	0.002	0.2211	0.08405	0.5816
Invasive blood pressure	0.025	0.1445	0.02652	0.7869
Interception	<0.001	0.0178	0.00514	0.0614

Source: Authors’ research. OR (Odds Ratio), IC (Confidence Interval). Model fit R^2^ Nagelkerke 0.631.

**Figure 2:**
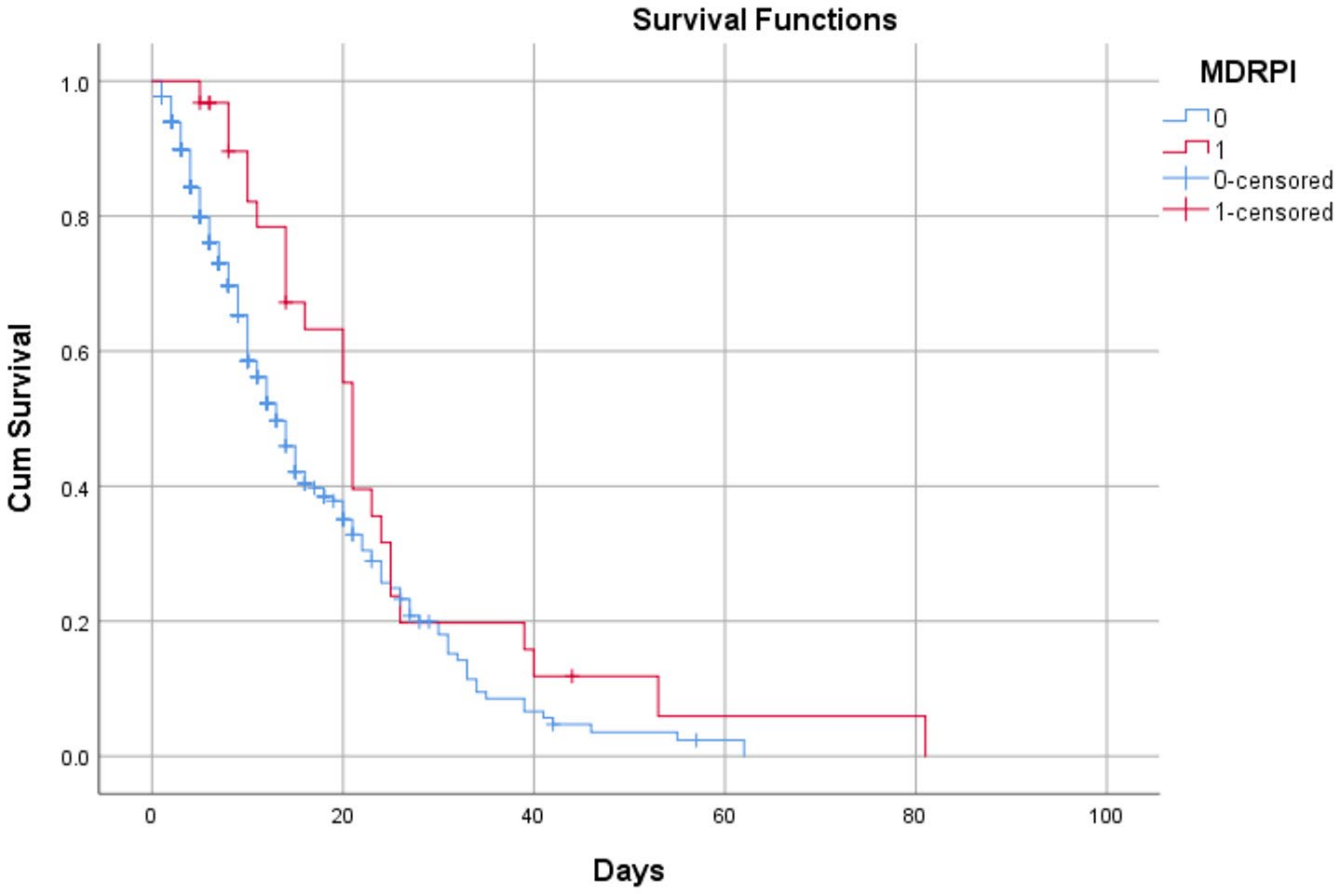
Survival analysis of patients with pressure injuries related to medical devices until the stage of discharge or death in patients admitted to an intensive care unit in a metropolitan region of the Amazon during the COVID-19 pandemic, Pará, Brazil, 2023. Source: Authors’ research. Statistical Package for the Social Sciences, 2023. Breslow (Generalized Wilcoxon) (0.007), Long hank (Mentel cox) (0.041), and Tarone-ware (0.011).

Survival analysis using Kaplan–Meier curves compared the time to discharge with the time of death among patients with MDRPI. The Breslow (generalized Wilcoxon) test showed a significant difference (*p* = 0.007), as did the log-rank (Mantel–Cox) test (*p* = 0.041) and the Tarone–Ware test (*p* = 0.011). Patients who were eventually discharged had a mean hospital stay of 17 days, whereas those who died remained hospitalized for a mean of 24 days. These findings indicate that MDRPI is associated with both prolonged hospitalization and increased mortality (Figure 2).

## 4 Discussion

This is the first retrospective cohort study conducted in the Amazon region to investigate factors associated with medical device-related pressure injuries (MDRPIs). It analyzed 109 numerical and categorical variables to characterize the profile of damage caused by medical devices in an intensive care unit. The study identified chronic obstructive pulmonary disease (COPD), use of orotracheal tubes (OTT), nasal catheters (NC), and length of hospital stay as risk factors. Conversely, systemic arterial hypertension (SAH), Braden scale score, and invasive arterial pressure monitoring were found to be protective predictors.

A study conducted in Nepal reported that hemodynamic instability and hypotension associated with comorbidities contribute significantly to MDRPI development among COPD patients (14). Additionally, a cross-sectional study involving 145 individuals with COPD demonstrated that disease exacerbations contribute to alveolar hypoxia, hypoxemia, and decreased oxygen saturation. These conditions weaken patients physically, increasing skin and tissue vulnerability (15). However, difficulties were encountered in locating robust literature specifically connecting COPD with MDRPI development. Further research is thus required to more clearly delineate risk factors contributing to MDRPI among COPD patients.

The existing literature consistently highlights medical device use as a risk factor for developing pressure injuries compared to patients without medical devices (16). In this study, OTT and NC emerged as significant predictors of MDRPI. Similarly, a prospective study conducted in Australia identified nasal catheters and endotracheal tubes as major causes of MDRPI, accounting for 21 and 13 cases, respectively, emphasizing their prevalence among ICU patients (17).

Length of hospital stay was also identified as a significant risk factor in this cohort. This finding aligns with the cross-sectional epidemiological study by Galetto et al. (9), in which prolonged hospitalization correlated significantly with MDRPI occurrence. Their study of 93 patients revealed that prolonged stays with therapeutic and monitoring devices increased MDRPI risk, resulting in extended hospitalization durations. This underscores the need for improved preventive strategies, especially in intensive care units.

Interestingly, systemic arterial hypertension was identified as a protective predictor in this study’s multivariate analysis. This result contradicts the prevailing literature, which typically characterizes pre-existing chronic conditions as intrinsic risk factors for pressure injury development in critically ill patients (18, 19). Cox and Roche (20), in their retrospective analysis of 306 ICU patients, reported hypotension and vasopressor use as notable risk factors for injury formation. There are currently no references suggesting that hypertension or vasodilator treatment is protective. We hypothesize that the protective effect identified here may be attributed to vasodilator treatment in hypertensive patients; however, this hypothesis remains untested in the current literature.

The Braden scale was also identified as a protective predictor in our retrospective cohort. The Braden scale is an established tool integrated into Nursing Care Systematization (NCS) practices for the prevention of pressure injuries (PI), assessing sensory perception, moisture, activity, mobility, nutrition, friction, and shear (21). Padula et al. (22) and Hanonu and Karadag (23) similarly demonstrated that lower Braden scores correlate significantly with higher MDRPI risk. Hanonu and Karadag specifically observed that patients with low Braden scale scores had a 1.81-fold increased risk of developing MDRPI in ICU settings.

Contrary to expectations, invasive arterial pressure (IAP) monitoring was also classified as protective against MDRPI. This finding diverges from previous studies, such as Reisdorfe’s (24) evaluation of ICU patients, where invasive arterial catheters were linked directly to injury formation. Reisdorfe observed that 8.5% of patients who used arterial punctures developed MDRPI, concluding that invasive monitoring devices present significant risks. Similarly, Ferreira et al. (25) highlighted the importance of nursing management in preventing MDRPIs associated with radial arterial catheters, which contrasts with the protective result observed in our cohort. Therefore, further research is needed to clarify this relationship.

The overall scarcity of studies specifically addressing MDRPI highlights the necessity for more detailed research. Further studies are essential to refine the classification systems recommended by the National Pressure Ulcer Advisory Panel (3) and accurately define causative and preventive factors.

Adherence to manufacturers’ guidelines for medical devices is essential for patient safety, treatment effectiveness, and MDRPI prevention. A retrospective qualitative study analyzing NOTIVISA data from 2007 to 2016 identified adverse events linked to inappropriate medical device handling by healthcare professionals, primarily due to insufficient knowledge or training (26, 27). Maia et al. (28) similarly highlighted systemic flaws in patient care, documenting 63,933 adverse events in NOTIVISA from 2014 to 2016, including 417 fatalities. These findings emphasize the critical importance of systematic reporting and improved healthcare practices.

In Brazil, studies addressing healthcare-related adverse events remain limited (28), particularly in the Amazon region, where incomplete patient records complicate accurate notification and reporting of such incidents. Strengthening notification systems within patient safety units is crucial to enhance preventive measures and patient care quality. Moreover, medical device quality significantly influences care outcomes. Previous qualitative-quantitative research indicates that high-quality medical devices are essential to prevent adverse events, minimize risks for patients and healthcare professionals, and avoid device-related failures (29).

The main limitation of this study is its retrospective design, relying exclusively on secondary data extracted from medical records. Such dependence introduces inherent risks affecting result validity, including incomplete, inaccurate, or illegible documentation and associated selection or classification biases. Furthermore, analyses were constrained by documented variables, preventing control for unrecorded confounding factors. These limitations, compounded by the small number of patients with MDRPI (*n* = 31) and a single-center setting during the COVID-19 pandemic, restrict statistical robustness and generalizability of the findings.

## 5 Conclusion

Of the 603 medical records analyzed, 62.85% of patients were female. The primary predictors of MDRPI identified in this study align closely with the existing literature. Specifically, chronic obstructive pulmonary disease, orotracheal tube use, nasal catheter use, and prolonged hospital stays emerged as significant risk factors, whereas systemic arterial hypertension, higher Braden scale scores, and invasive arterial pressure monitoring were identified as protective factors.

This cohort did not exhaustively address all aspects associated with MDRPI. Therefore, future prospective studies are strongly recommended. The limited literature available in Brazil highlights an urgent need for further research to strengthen evidence-based strategies for preventing MDRPI in intensive care settings, thus positively impacting patient safety, care quality, and reducing mortality.

As the first study of its kind in our region, it confirmed that local risk factors for MDRPI are consistent with global findings, despite the greater vulnerability related to social and health determinants. Improving nursing care and working conditions in ICUs is essential to mitigate MDRPI risk. The findings presented here can guide continuous professional development for nursing teams and inform educational discussions within academic nursing programs.

## Data Availability

The original contributions presented in the study are included in the article/supplementary material, further inquiries can be directed to the corresponding author.
